# Cardiac Troponin I in Non- Acute Coronary Syndrome Patients with Chronic Kidney Disease

**DOI:** 10.1371/journal.pone.0082752

**Published:** 2013-12-12

**Authors:** Shanying Chen, Chunhong Huang, Bide Wu, Xuejian Lian, Xuqiao Mei, Jianxin Wan

**Affiliations:** 1 Department of Nephrology, Zhangzhou Affiliated Hospital of Fujian Medical University, Zhangzhou, China; 2 Clinical Laboratory, Zhangzhou Affiliated Hospital of Fujian Medical University, Zhangzhou, China; 3 Department of Nephrology, the First Affiliated Hospital of Fujian Medical University, Fuzhou, China; S.G.Battista Hospital, Italy

## Abstract

**Objective:**

The aim of this study was to assess the results of troponin I (cTnI) in non- acute Coronary Syndrome (ACS) patients with chronic kidney disease (CKD). We also examined the risk factors for elevated cTnI in non-ACS patients with CKD and whether stage 5 CKD modifies the associations of elevated cTnI and the risk factors in non-ACS patients with CKD.

**Methods:**

A retrospective study was performed. Logistic regression models were used.

**Results:**

293 non-ACS patients with CKD were included in the current study. 43.34% non-ACS patients with CKD have an elevated cTnI level and 5.12% have an elevated cTnT level in MI range. In CKD patients without ACS and heart failure, only 26.03% (38/146) patients have an elevated cTnT level. In adjusted analyses, age, diastolic blood pressure and congestive heart failure is associated with an elevated cTnI level in non-ACS patients with CKD. Congestive heart failure is associated with an elevated cTnI level in non-ACS patients with CKD (OR 2.30, 95% CI 1.08,4.88, P=0.03). Stage 5 CKD does not modify the association of congestive heart failure and an elevated cTnI level.

**Conclusion:**

43.34% non-ACS patients with CKD and 26.03% CKD patients without ACS and congestive heart failure have an elevated cTnI level. Congestive heart failure is associated with an elevated cTnI level in non-ACS patients with CKD. Stage 5 CKD does not modify the association of congestive heart failure and an elevated cTnI level.

## Introduction

Cardiac troponin T (cTnT) and cardiac troponin I (cTnI) have been shown to be highly sensitive and specific markers of myocardial cell injury [1,2]. But previous studies have indicated that cTnT may be elevated in patients with chronic renal failure in the absence of ischemic heart disease [3]. However, there is a paucity of data on cardiac troponin I (cTnI) measured by a high sensitivity assay in CKD patients without detectable acute coronary syndromes (ACS).

The aim of this study was to assess the results of cTnI in non-ACS patients with CKD. We also examined the risk factors for elevated cTnI in non-ACS patients with CKD and whether stage 5 CKD modifies the associations of elevated cTnI and the risk factors in non-ACS patients with CKD.

## Methods and Patients

### Patients

This study was approved by the ethics committee of the Zhangzhou Affiliated Hospital of Fujian Medical University. The ethics committee of the Zhangzhou Affiliated Hospital of Fujian Medical University waived the need for written informed consent from the participants. A retrospective study was performed. We included in patients with CKD [4] in Zhangzhou Affiliated Hospital of Fujian Medical University between January of 2009 and December of 2011, and in whom cTnI was determined. Our exclusion criteria included 1) chest pain, and 2) ST segment elevation or pathologic Q wave formation in electrocardiogram (ECG). Variables included in the study were: age, gender, smoking history, diabetes mellitus, hypertension, blood pressure, congestive heart failure, primary renal disease, determination of serum cTnI, CK-MB, serum calcium-phosphorus product, serum C-reactive protein (CRP), serum low density lipoprotein (LDL), serum high density lipoprotein (HDL), serum homocysteine, hemoglobin, platelet, 12-lead ECG and cardiothoracic ratio determined by x- ray. All patients had evidence of kidney damage (eGFR <60 mL/min/1.73 m^2^ or proteinuria ) at least 3 months. 

According to the concentrations of cTnI, patients were divided into two groups: 1) patients with normal cTnI levels (0-0.06 ng/ml), 2) patients with elevated cTnI levels(> 0.06 ng/ml).

### Clinical and laboratory variables

Venous blood samples were collected in evacuated tubes. The samples were centrifuged at 3500 rpm for 5 min. Samples were analyzed for cTn I, CK-MB, blood urea nitrogen, LDL, HDL, creatinine, calcium, phosphorus, CRP, LDL, HDL, homocysteine, and parathyroid hormone (PTH).

 High sensitivity cardiac troponin-I (TnIc) was measured with the ADVIA Centaur assay (Siemens healthcare diagnostics Inc), which is a direct chemical luminescence immunoassay method with two antibodies. An increased cTn concentration is defined as a value exceeding the 99th percentile of a normal reference population. According to the manufacturers, the population reference value is less than 0.06 ng/ml, and the cut-off point considered for diagnosing AMI is 0.6 ng/ml [5]. 

The mass concentration of CK-MB was measured by Hitachi AU5400, which uses an immunosuppression method. The reference value used is < 25 u/l. Creatinine was measured by Hitachi AU5400, using an oxidase method. Glomerular filtration rate was estimated using the four-variable Modification of Diet in Renal Disease (MDRD) equation [175× (Scr)^-1.234^ × (Age)^-0.179^ ×(if female, ×0.79) ] [6]. High sensitivity C-reactive protein (CRP) was measured using enzymatic immunoassay turbidimetric method. 

 Data on age, sex, current or past cigarette smoking, primary disease, blood pressure, personal history and family history, and congestive heart failure were obtained from health records and discharge reports. Urine volumes refer to the average amount of two consecutive days urine output (in patients receiving hemodialysis, two consecutive days included a non-dialysis treatment day and a dialysis day.) 

Patients received a standard 12-lead resting electrocardiograph (ECG), using a Nalong Medical System.

Transthoracic echocardiography was performed using a Sequoia 512 ultrasonic diagnostic apparatus (Siemens, Inc). Posterior left ventricular wall thickness, interventricular septal thickness and ejection fraction were obtained from reports. 

### Definitions

CKD was defined as eGFR less than 60 ml/min/1.73m^2^ and/or proteinuria ≥1g/24hours [4] . The stages of CKD were based on the eGFR. Stage 1: kidney damage with normal or relatively high GFR (≥90 mL/min/1.73 m^2^); Stage 2: Mild reduction in GFR (60–89 mL/min/1.73 m^2^) with kidney damage.Stage; 3: Moderate reduction in GFR (30–59 mL/min/1.73 m^2^); Stage 4:Severe reduction in GFR (15–29 mL/min/1.73 m^2^); Stage 5 :GFR <15 mL/min/1.73 m^2^, or permanent renal replacement therapy [4] . The ECG criteria for left ventricular hypertrophy (LVH) included voltage criteria ( any of ) R in V_5_ ≥ 25mm, S in V_1_ + R in V_5_ > 40 mm (men), and S in V_1_ + R in V_5_ > 35 mm (women) and ST-T abnormalities [7]. Hypoalbuminemia was defined as serum albumin < 35g/l. An intermediated elevated CK-MB level was defined as CK-MB > 25 u/l and < 50 u/l. 

### Statistical analyses

Data were analyzed using Stata (version 11). Continuous variables were shown as mean ± standard deviation for normal distribution variables. Median and interquartile range were used to show skewed distributed continuous variables. The categorical variables were presented as absolute and relative (%) values. A two-tailed p value <0.05 was considered significant. 

Baseline characteristics of patients were examined in both normal cTnI level and elevated cTnI level subgroups. Baseline characteristics of two subgroups were examined. Student’s t test or Wilcoxon rank-sum test was used for continuous variables and the chi-squared test or Fisher’s exact test was used for categorical variables. CKD was categorized as: stage 1-3, stage 4, and stage 5.

To examine the risk factors associated with an elevated cTnI level in non-ACS patients with CKD, logistic regression models were used. Firstly, variables were examined in unadjusted model. These variables included: demographic characteristics (age and sex); clinical characteristics (diabetes, hypertension, systolic and diastolic blood pressure, LDL, HDL, current smoking, and coronary artery disease [prior myocardial infarction or revascularization]) . According to previous studies, anemia and pathologic LVH might be associate with troponin [8]. Hemoglobin, ECG diagnosis of LVH, cardiothoracic ratio determined by x- ray, posterior left ventricular wall thickness, interventricular septal thickness determined by echocardiography and ejection fraction were also included in analyses. We also examine whether some other important variables in CKD patients are associated with an elevated troponin level. These variables are serum albumin, serum calcium-phosphorus product, urine volume and stage 5 CKD. Next variables were examined in adjusted model. Finally, to examine whether stage 5 CKD modifies the association of risk factors and an elevated cTnI level, stage 5 CKD was added to the model. 

All variables with skewed distributions were logarithmically transformed before being analyzed. 

## Results

During the study period, cTnI determinations were done for 368 patients in the department of nephrology, of which 12 patients were diagnosed as acute kidney injure, 12 patients had no CKD, and other 51 patients were excluded because of chest pain or ST segment elevation or pathologic Q wave formation in ECG. 293 non-ACS patients with CKD were included in the current study. 

### Patient Characteristics

161 (54.95%) were men and 132 (45.05%) were women. The age range was 20 to 89 years old (mean, 60.18 years). 127 (43.34%) patients had an elevated cTnI level (> 0.06 ng/ml). The characteristics of the patients were showed in the table 1. 

**Table 1:** Baseline characteristics ^***a***^ of patients according to troponin I levels.

	Entire Cohort	**Troponin ≤ 0.06 ng/ml**	**Troponin > 0.06 ng/ml**	**P value**
	n = 293	n = 166	n = 127	
**Demographics**				
Age (Years)	60.18 ± 14.73	62.31 ± 14.28	57.39 ± 14.91	0.004
Male (%)	161 (54.95)	93 (56.02)	68 (53.54)	0.67
**Clinical Characteristics**				
History of myocardinal infarction (%)	15 (5.12)	7 (4.22)	8 (6.23)	0.42
History of Diabetes (%)	103 (35.15)	55 (33.13)	48 (37.80)	0.41
Hypertension (%)	226 (78.77)	123 (74.54)	103 (84.25)	0.04
Primary disease (glomerulonephritis)	49 (16.72)	29 (17.46)	20 (15.75)	0.74
Primary disease (diabetes)	78 (26.62)	40 (26.14)	38 (27.14)	0.40
Primary disease (hypertension)	49 (16.72)	33 (19.88)	16 (12.60)	0.16
Current smoker (%)	64 (21.84)	34 (20.48)	30 (24.59)	0.52
Systolic blood pressure (mmHg)	161.34 ± 39.39	156.01 ± 37.38	168.31 ± 41.00	0.008
Diastolic blood pressure (mmHg)	91.00 ± 22.63	87.52 ± 19.40	95.54 ± 25.	0.003
**Laboratory**				
Serum creatitine (umol/l)	682.53 ± 455.08	561.12 ± 415.80	841.23 ± 456.86	<0.001
Serum uric acid (umol/l)	496.93 ± 187.52	455.00 ± 156.99	552.94 ± 209.76	<0.001
Estimated glomerular filtration rate ( ml/min/1.73m^2^)	21.48 ± 35.91	28.70 ± 40.40	12.03 ± 26.31	<0.001
CKD				<0.001
CKD (stage 5 )	218 (74.40)	108 (65.06)	110 (86.61)	
CKD (stage 4 )	23 (13.86)	17 (10.24)	6 (4.72)	
CKD (stage 1 - 3)	52 (17.75)	41 (24.70)	11 (8.66)	
Bicarbonate (mmol/l)	19.82 ± 6.87	20.90 ± 6.66	18.42 ± 6.91	0.002
Proteinuria	N = 265	N= 160	N=105	0.04
Negetive	43 (16.23)	29 (18.13)	14 (13.33)	
trace	18 (6.79)	16 (10.00)	2 (1.90)	
1 +	42 (15.85)	26 (16.25)	16 (15.24)	
2+	73 (27.55)	45 (28.13)	28 (26.67)	
3 +	79 (29.81)	39 (24.38)	40 (38.10)	
4 +	10 (3.77)	5 (3.13)	5 (4.76)	
Albumin (g/L)	33.17 ± 7.05	33.50 ± 7.29	32.72 ± 6.72	0.36
Hemoglobin (g/l)	92.74 ± 28.24	93.81 ± 26.27	91.33 ± 26.88	0.46
Calcium-phosphorus product(mmo/l . mmol/l)	4.08 (3.04 - 5.47)	3.80 (2.89 - 4.68)	4.57 (3.40 - 6.14)	0.002
Platelet (x 10 9/l)	217.27 ± 94.63	221.31 ± 95.72	206.14 ± 91.13	0.16
Serum C-reactive protein (mg/l)	N = 150	N = 91	N = 59	
	6.75 (1.89 - 25.2)	4.07 (1.22 - 16.00)	13.30 (3.12 - 37.20)	0.001
Serum triglyceride (mmol/l)	1.22 (0.84 - 1.93)	1.22 (0.82 - 1.94)	1.26 (0.90 - 1.99)	0.35
Serum low density lipoprotein (mmol/l)	2.99 ± 1.81	3.07 ± 1.96	2.87 ± 1.60	0.38
Serum high density lipoprotein (mmol/l)	1.06 ± 0.41	1.09 ± 0.45	1.02 ± 0.34	0.2
Troponin I (ng/ml)	0.05 (0.02 - 0.15)	0.02 (0.01 - 0.04)	0.16 (0.09 - 0.28)	<0.001
Creatine kinase - MB(mmol/l)	17 (10.00 - 30.00)	13.95 (8.00 - 26.00)	25.30 (13.70 - 33.00)	<0.001
Normal	188 (64.16)	122 (73.49)	66 (51.97)	<0.001
Intermediated elevated	77 (26.28)	32 (19.28)	45 (15.36)	
Elevated	28 (9.56)	12 (7.23)	16 (12.60)	
Homocysteine	19.5 (12.60 - 27.30)	18.20 (11.60 - 26.40)	21.45 (14.80 - 27.90)	0.06
Fibrinogen	4.28 ± 1.69	4.08 ± 1.27	4.54 ± 2.11	0.03
ECG diagnosis of left ventricular hypertrophy	60 (20.48)	26 (15.66)	34 (26.77)	0.02
Cardiothoracic ratio > 0.5	92 (31.40)	36 (21.69)	56 (44.09)	<0.001
Posterior left ventricular wall thickness (mm)	N = 91	N = 57	N = 34	
	8.4 (6.90 - 9.60)	8.1 (6.90 - 9.10)	8.6 (7.30 - 10.80)	0.07
Ejection fraction (%)	57.7 (53.20 - 62.1)	57.7 (54.5 - 61.04)	56.5 (51.64 - 62.6)	< 0.001
Interventricular septal thickness (mm)	9.4 (7.6 -10.4)	10.3 (7.39 - 11.90)	9.6 (7.5 - 11.30)	0.14
Intact parathyroid hormone (pg/ml)	N =133	N = 71	N = 62	
	228 (118.6 - 392.1)	199.9 (109.4 - 372.8)	262.65 (121.6 - 395.8)	0.12
Urine volume (ml/24hours)	1020 (500 - 1700)	1400 (800 - 1700)	800 (200 - 1600)	<0.001
Hemodialysis	145 (49.49)	63 (34.95)	82 (64.57)	<0.001
Peritoneal dialysis	26 (8.87)	14 (9.15)	12 (8.57)	0.76

a Mean ± SD or median (25th to 75th percentiles) for continuous variables and absolute and relative (%) values for category variables are presented.

In the entire cohort, 218 subjects had stage 5 CKD. Among these patients, 145 patients received hemodialysis and 26 patients received peritoneal dialysis. 23 patients had stage 4 CKD and 52 had stage 1-3 CKD. Patients with an elevated troponin level had significantly higher systolic blood pressure, diastolic blood pressure, higher serum creatitine, higher serum uric acid, lower eGFR, higher calcium-phosphorus product, and higher CRP. Patients with an elevated troponin level also had higher prevalence of LVH. 12 patients had atrial fibrillation, and one patient had superventricular tachycardia. Patients with an elevated troponin level had lower ejection fraction than patients with an normal troponin level.

### Concentration of cTnI

The range of cTnI concentrations was 0-3.67ng/ml with a median value of 0.05 ng/ml. Among 293 patients, 166 (56.66%) patients had a normal cTnI level (< 0.06 ng/mL), and 112 patients (38.23 %) had an elevated cTnI level (concentration 0.06-0.6ng/ml), and 15 patients (5.12%) had an elevated cTnT level concentration > 0.6 ng/mL. 

### Interpretation of an elevated cTnI level in non-ACS patients with CKD

Among 127 patients with an elevated cTnI level, 110 patients had stage 5 CKD, and 82 patients received hemodialysis, and 12 patients received peritoneal dialysis. 112 patients with an elevated cTnI level had congestive heart failure. 6 patients with an elevated cTnI level had atrial fibrillation, and one patient had superventricular tachycardia. No patients received percutaneous coronary intervention, coronary artery bypass graft surgery, cardiac surgery, and electrical shock. No patients had chest wall injure.

### Association of an elevated cTnI level and CK-MB in non-ACS patients with CKD

Among 166 patients with a normal cTnI level, 122 patients had a normal CK-MB level, and 32 patients had an intermediated elevated CK-MB level, and 12 patients had an elevated CKMB level (> 50 u/l). Among 127 patients with an elevated cTnI level, 66 patients had a normal CK-MB level. Only 61 patients had both an elevated cTnI level and an elevated CK-MB level, and 122 patients had both a normal cTnI level and a normal CK-MB level. Univariate regression model showed that log CK-MB is associated with log cTnI (coef 0.38, 95% CI 0.21, 0.54. P < 0.001. not showed in the table). 

### Risk factors for an elevated cTnI level in non-ACS patients with CKD (table 2)

**Table 2:** Association of variables with an elevated troponin I level.

	**Model - 1** ^a^		**Model - 2** ^b^		**Model - 3** ^c^	
	**OR (95% CI)**	**P value**	**OR (95% CI)**	**P value**	**OR (95% CI)**	**P value**
Age	0.98 (0.96, 0.99)	0.005	0.97 (0.95, 1.00)	0.03	0.98(0.95, 1.00)	0.047
Men	0.9 (0.57, 1.44)	0.67	0.83 (1.10, 3.15)	0.6	0.85 (0.42, 1.70)	0.65
Diabetes	1.23 (0.76, 1.99)	0.41	1.27 (0.62, 2.63)	0.51	1.29 (0.62, 2.69)	0.49
Hypertension	1.83 (1.01, 3.30)	0.046	1.31 (0.51, 3.38)	0.58	1.2 (0.49, 3.18)	0.72
Current Smoker	1.2 (0.69, 2.09)	0.64	1.53 (0.70, 3.36)	0.29	1.54 (0.70, 3.38)	0.29
History of myocardial infarction	1.53 (0.54, 4.33)	0.43	2.19 (0.55, 8.62)	0.26	2.37 (0.59,9.56)	0.23
Congestive heart failure	4.45 (2.39, 8.31)	< 0.001	2.43 (1.35, 5.13)	0.02	2.3 (1.08,4.88)	0.03
Low density lipoprotein	0.94 (0.82, 1.08)	0.38	1.12 (0.91, 1.38)	0.27	1.14 (0.93,1.41)	0.21
High density lipoprotein	0.67 (0.36, 1.23)	0.2	0.5 (0.19, 1.38)	0.17	0.53 (0.19, 1.38)	0.19
ECG diagnosis of left ventricular hypertrophy	1.98 (1.11, 3.53)	0.02	1.42 (0.65, 3.11)	0.38	1.41 (0.34, 3.09)	0.4
Cardiothoracic ratio > 0.5	3.03 (1.79, 5.12)	< 0.001	1.75 (0.86, 3.56)	0.12	1.79 (0.88, 3.64)	0.11
CKD 5	3.47 (1.90, 6.35)	< 0.001	--		1.68 (0.68, 4.15)	0.26
Systolic blood pressure	1.01 (1.00, 1.01)	0.009	0.99 (0.98, 1.01)	0.26	0.99 (0.97, 1.00)	0.18
Diastolic blood pressure	1.02 (1.01, 1.03)	0.003	1.03 (1.00, 1.05)	0.04	1.03 (1.00, 1.06)	0.031
Urine volumn	0.74 (0.58, 0.95)	0.02	0.8 (0.58, 1.11)	0.19	0.82 (0.59, 1.14)	0.24
Calcium-phosphorus product	3.06 (1.68, 5.58)	< 0.001	1.45 (0.65, 3.21)	0.37	1.21 (0.52, 2.85)	0.66
Intact parathyroid hormone	1.2 (0.86, 1.68)	0.28				
Hypoalbuminemia	1.16 (0.73, 1.85)	0.53	1.30 (0.66, 2.56)	0.45	1.28 (0.65, 2.54)	0.48
Posterior left ventricular wall thickness	3.36 (0.57, 19.83)	0.18	--	--	--	--
Interventricular septal thickness	5.55 (0.82, 37.79)	0.08	--	--	--	--
Ejection fraction	1.11 (0.11, 11.63)	0.93	--	--	--	--

^c^ Unadjusted

^b^ Adjusted for age, sex, hypertension, history of myocardial infarction, congestive heart failure, current smoker, systolic blood pressure, diastolic blood pressure, high density lipoprotein cholesterol, low density lipoprotein cholesterol, LVH, cardiothoracic ratio > 0.5, Hypoalbuminemia, log serum calcium-phosphorus product, log urine volumn

^c^ Adjusted for above + stage 5 CKD

In unadjusted analyses, age, blood pressure, urine volume, serum calcium-phosphorus product, and congestive heart failure are associated with an elevated cTnI level in non-ACS patients with CKD. Compared to stage 1-4 CKD, patients with stage 5 CKD had higher cTnI levels. In unadjusted analyses, PTH, posterior left ventricular wall thickness, interventricular septal thickness and ejection fraction were not associated with an elevated cTnI level.

 Only 91 patients had data on echocardiography and 150 patients have received CRP test and 133 patients have received PTH test, so posterior left ventricular wall thickness, interventricular septal thickness, ejection fraction, CRP, and PTH were not included in adjusted model. In adjusted analyses, only age, diastolic blood pressure and congestive heart failure are associated with an elevated cTnI level in non-ACS patients with CKD. Congestive heart failure was significantly associated with an elevated cTnI level in non-ACS patients with CKD (OR 2.43, 95% CI 1. 35, 5.13, P=0.02). Further adjustment for stage 5 CKD had little impact on the odd ratios, and the odd ratios were 2.30( 95% CI 1.08, 4.88, P=0.03) . Stage 5 CKD does not modify the association of congestive heart failure and an elevated cTnI level.

### cTnI levels in patients without congestive heart failure

Among 146 patients without congestive heart failure, 73.97% (108) patients had a normal cTnI level, with median 0.03 (0.01, 0.07). Among 38 patients with an elevated cTnI level, 11 had ECG diagnosis of LVH, 6 had cardiothoracic ratio > 0.5. 

## Discussion

In the current study, among 293 non-ACS patients with CKD, 166 (56.66%) patients had a normal cTnI level and 112 patients (38.23 %) had an elevated cTnI level (concentration 0.06-0.6ng/ml), and 15 patients (5.12%) had an elevated cTnT level in MI range. In 146 patients without congestive heart failure, only 26.03% (38/146) patients had an elevated cTnI level. 122 patients had both a normal cTnI level and a normal CK-MB level and 61 patients had both an elevated cTnI level and an elevated CK-MB level. The results of our study suggest that congestive heart failure is associated with an elevated cTnI level in non-ACS patients with CKD. Stage 5 CKD does not modify the association of congestive heart failure and an elevated cTnI level. 

Troponin C, troponinI and troponin T are all present in both cardiac and skeletal muscle. Cardiac troponin T and cTnI are derived from genes that are specific to the heart [9]. Since the introduction of troponin testing in the early 1990s, there have been questions about the elevated level troponin in CKD patients [10,11,12]. Interpretation of cardiac biomarkers, particularly cardiac troponin in the setting of CKD, has been controversial [11]. Troponin T and cTnI are proteins with molecular mass of 37 kDa and 22 kDa and it is improbable that the kidneys are primarily responsible for their clearance from the serum [10,13,14]. Elevated troponins, particularly cTnT, have been observed in patients with various degrees of renal failure and treatment modalities in the absence of an acute coronary event [14,15]. The possibility that increased troponin levels reflect decreased clearance, or analytical interference from uremic serum, may not have been excluded. 

Troponin T is fragmented into molecules small enough to be cleared by the kidneys of healthy subjects. Impaired renal function causes accumulation of these cTnT fragments and is very likely the cause of the unexplained elevations of serum cTnT found in patients with severe renal failure[3]. However, it has not been demonstrated that the fragments will cross-react in the Roche cTnT assay [3]. Compared with cTnI, troponin T is more frequently increased in asymptomatic patients with ESRD [16] Although troponin T was cardiac specific, cross-reaction between cardic troponin T and skeletal troponin T might lead to false postive [17]. Even a new-generation assay using cardiac specific capture and detection antibodies was introduced , cross-reactivity between cardiac troponin T and skeletal troponin T is still present [18] . cTnI is not expressed in skeletal muscle or other tissues or in response to degenerative or regenerative muscle disease processes. The skeletal muscle regenerates after damage might cause the muscle revert back to expressing its embryonic forms of cardiac Troponin T (cTnT) but no cTnI [19]. In response to fasting, acidosis, uncontrolled diabetes mellitus, or sepsis, muscle protein breakdown increases in patients with kidney diease[20]. Accelerated muscle protein degradation might also be a pausible explanation for the inconsistency detection between troponin T and cTnI in renal failure patients with absence of an acute coronary event.

Although early cardiac troponin assays were considered as a replacement test for CK - MB measurement, equivalence between the markers could not be demonstrated. In 12% to 39% of patients who were negative for CK-MB, cardiac troponin results were positive[10]. In the current study, among 188 patients with a normal CK-MB level, 66 (35.10%) patients had an elevated cTnI level. These data raised the question as to whether discordant troponin and CK-MB results were falsely positive or indicative of a more sensitive test that classified patients more accurately [10]. In the current study, only 166 (56.66%) non-ACS patients with CKD had a normal level cTnI. Among 127 CKD patients with an elevated cTn I level, 88.19% (112) had congestive heart failure. The results suggest that troponin might be more sensitive in patients with heart failure. In the large multi-center database, nearly 6.2% patients with heart failure had abnormal troponin test [10]. The results were based on the analyses excluding patients with serum creatinine > 2.0 mg/dl. In the current study, 60.54% patients with congestive heart failure had abnormal cTnI test. The elevated troponin level might suggest the underlying myocardial injury in CKD patients with heart failure.

The presence of cardiac troponin in blood indicated that cardiac injury had occurred. The high-sensitivity assays remarkably increased sensitivity and increased early detection of myocardial necrosis, but this was associated with decreased specificity [10]. With the highly sensitive cTnT (hs-cTnT) and assay, it is possible to detect circulating cTnT in virtually all patients with chronic coronary artery disease or congestive heart failure; moreover, 25% to 67% of adults from the general population have detectable troponin levels with this assay [21]. In the Dallas Heart study, higher cTnT was independently associated with magnetic resonance imaging-defined measures of abnormal cardiac structure and function, such as left ventricular (LV) hypertrophy and LV dysfunction, but no independent association was seen with coronary artery calcium, a measure of atherosclerosis [22]. In 2 large population-based studies, the ARIC (Atherosclerosis Risk in Communities) study and the Cardiovascular Health Study, associations with cTnT were much stronger for incident heart failure than for MI [21]. Recent studies have indicated that circulating cTnT levels in patients with CKD are predominantly an indicator of pathologic LV hypertrophy [23]. In the current study, it is also indicated that congestive heart failure is independently associated with an elevated cTnI level. The results of the current study support that an elevated cTnI level in non-ACS patients with CKD is an indicator for underlying myocardial injury in patients with heart failure.

In the current study, among 146 patients without congestive heart failure, 26.03% (38) patients had an elevated cTnI level. Previous study showed that cTnI levels were increased in 5–18% of asymptomatic hemodialysis patients with standard assays [16,24]. But in a recent study, newer-generation, high-sensitivity assays were used, 37% asymptomatic hemodialysis patients had an elevated cTnI level. It was found that higher cTnI levels at baseline were associated with a history of coronary heart disease, LVH, lower ejection fraction and higher serum phosphate levels. But in this study, the sample size was small, and only 51patients were included [25]. 

Previous studies based on CKD patients suggested that troponin T was associated with left ventricular structure and function in CKD [23]. In the current study, patients with an elevated troponin level had lower ejection fraction and the difference was significant. And patients with an elevated troponin level also had higher posterior left ventricular wall thickness and the difference was borderly significant (p=0.07). But only 91 patients had data on echocardiography. As we have mentioned above, the inconsistency detection between troponin T and cTnI had been found in renal failure patients. The associations of cTnI with left ventricular structure and function in CKD patients need to be further studied in a bigger sample. 

In multivariate model, stage 5 CKD is not significantly associated with an elevated cTnI level. Although there are some data to suggest that residual renal function may affect troponin levels [26,27]. the results of the current study suggest that urine volume is not associated with an elevated cTnI level. It is unlikely that kidney failure is responsible for an elevated cTnI level. 

Chronic hyperglycemia is commonly believed to contribute to premature cardiovascular disease morbidity and mortality. Diabetes has long been implicated as a predisposing factor for heart failure [21]. But in the current study, we failed to find the association of diabetes with an elevatedcTnI level. The concept of a specific “diabetic cardiomyopathy” has been more controversial [21]. 

Vascular calcification is now recognized as a strong predictor of cardiovascular events in the general population as well as diabetic and end-stage renal disease patients. High total body burden of calcium and phosphorus may contribute to vascular calcification in CKD patients [24,28]. In the current study, we also included serum calcium-phosphorus product in analyses. The results suggest that serum calcium-phosphorus product is not associated with an elevated cTnI level.

The first limitation of this study is a retrospective, single-centre study and lack of longitudinal data. Previous study has suggested prognostic value of troponin in patients with chronic kidney disease [16,29,30,31]. Second, only 91 patients have received echocardiography, and we did not included echocardiography in adjusted model. Recently study indicated that detectable cTnT had a strong association with LV hypertrophy [23]. In the current study, posterior left ventricular wall thickness and interventricular septal thickness were not associated with an elevated cTnI level presumably reflecting the small sample size. But compared with echocardiography, ECG is cheaper and more accessible. Third, troponin level might be reduced by hemodialysis [32], but we did not assess the association of dialysis and cTnI levels. 

## Conclusion

The results of the current study suggest that 43.34% non-ACS patients with CKD have an elevated cTnI level and 5.12% have an elevated cTnT level in MI range. In CKD patients without ACS and congestive heart failure, only 26.03% (38/146) patients have an elevated cTnT level. Congestive heart failure is associated with an elevated cTnI level in non-ACS patients with CKD. Stage 5 CKD does not modify the association of congestive heart failure and an elevated cTnI level. 
